# Determinants of preventable readmissions in the United States: a systematic review

**DOI:** 10.1186/1748-5908-5-88

**Published:** 2010-11-17

**Authors:** Joshua R Vest, Larry D Gamm, Brock A Oxford, Martha I Gonzalez, Kevin M Slawson

**Affiliations:** 1 Jiann-Ping Hsu College of Public Health, Georgia Southern University Hendricks Hall, PO Box 8015, Statesboro, GA 30460-8015, USA; 2 Texas A&M Health Science Center, School of Rural Public Health, Department of Health Policy & Management, 1266 TAMU, College Station, TX 77843, USA; 3 Texas A&M University, Dwight Look College of Engineering, Department of Industrial & Systems Engineering. 241 Zachry Engineering Research Center, Texas A&M University, 3131 TAMU, College Station, TX 77843-3131, USA

## Abstract

**Background:**

Hospital readmissions are a leading topic of healthcare policy and practice reform because they are common, costly, and potentially avoidable events. Hospitals face the prospect of reduced or eliminated reimbursement for an increasing number of preventable readmissions under nationwide cost savings and quality improvement efforts. To meet the current changes and future expectations, organizations are looking for potential strategies to reduce readmissions. We undertook a systematic review of the literature to determine what factors are associated with preventable readmissions.

**Methods:**

We conducted a review of the English language medicine, health, and health services research literature (2000 to 2009) for research studies dealing with unplanned, avoidable, preventable, or early readmissions. Each of these modifying terms was included in keyword searches of readmissions or rehospitalizations in Medline, ISI, CINAHL, The Cochrane Library, ProQuest Health Management, and PAIS International. Results were limited to US adult populations.

**Results:**

The review included 37 studies with significant variation in index conditions, readmitting conditions, timeframe, and terminology. Studies of cardiovascular-related readmissions were most common, followed by all cause readmissions, other surgical procedures, and other specific-conditions. Patient-level indicators of general ill health or complexity were the commonly identified risk factors. While more than one study demonstrated preventable readmissions vary by hospital, identification of many specific organizational level characteristics was lacking.

**Conclusions:**

The current literature on preventable readmissions in the US contains evidence from a variety of patient populations, geographical locations, healthcare settings, study designs, clinical and theoretical perspectives, and conditions. However, definitional variations, clear gaps, and methodological challenges limit translation of this literature into guidance for the operation and management of healthcare organizations. We recommend that those organizations that propose to reward reductions in preventable readmissions invest in additional research across multiple hospitals in order to fill this serious gap in knowledge of great potential value to payers, providers, and patients.

## Introduction

Preventable hospital readmissions possess all the hallmark characteristics of healthcare events prime for intervention and reform. First, readmissions are costly: estimated at $17 billion annually to the Medicare program for unplanned readmissions [1] and at nearly $730 million for preventable conditions in four states within just six months [2]. Second, readmissions to the hospital within a relatively short span of time are common among the total population [3], Medicare patients [1,4], veterans [5], and preterm infants [6], underscoring the pervasiveness of the problem across hospitals. Third, disparities in readmission rates exist by race, ethnicity, and age [2]. Last, the idea of the unplanned, early, or preventable readmission is historically viewed as the result of quality shortcomings or system failures [7].

As common, costly, and potentially avoidable events, it is not surprising that hospital readmissions are a leading topic of practice reform and healthcare policy. Payers in the US have explored readmission rates as measures of quality for decades [8]. Today, the Hospital Quality Alliance [9], a consortium of payers, healthcare organizations, and regulators, includes readmission rates for select inpatient conditions as quality indicators, and the Institute for Healthcare Improvement [10] also promotes readmission rate a quality measure. Likewise, the Department of Health and Human services [11] provides selected readmission rates as part of Hospital Compare's efforts to 'promote reporting on hospital quality of care' and Thomson Reuters uses the measure in their annual 100 Top Hospitals List [12]. The Obama administration has identified reducing readmissions as a cost savings mechanism to finance reform efforts [13]. The Centers for Medicare and Medicaid Services recommended reducing payments for readmissions [14] and along with the National Quality Forum, has already defined some readmission as truly preventable and therefore not worthy of reimbursement [15]. Joining this call for reducing preventable readmissions is the growing interest in bundled payments and accountable care organizations as means to improve healthcare quality and efficiency. These approaches may reduce preventable readmissions by creating episodes of care, which encompass a significant portion of patients' pre- and post-hospital care periods [16].

However, for healthcare organizations, particularly hospitals and hospital systems, these changes and interest in readmissions are viewed as a harbinger of more uncompensated services and care [17]. To meet the current challenges and future expectations, organizations are looking for potential strategies, within and without the hospital, to reduce such preventable readmissions [18]. Aligning hospital operations and management practices with the desired goal of reduced preventable readmissions requires the identification of modifiable risk factors regarding patients and care. In light of these challenges, needs, and increasing pressure for a systemic response to preventable readmissions, we undertook a systematic review of the literature to determine how the existing literature defined preventable readmissions in terms of index condition, reasons for readmission, and timeframe, and what factors are associated with preventable readmissions. Without clear answers to these questions, valid and objective criteria for measuring preventable readmissions are likely to be in short supply and evidence-based strategies that might be used by providers to reduce such readmissions will be significantly delayed.

### Conceptual framework

For the purposes of this review, we consider a preventable readmission as an unintended and undesired subsequent post-discharge hospitalization, where the probability is subject to the influence of multiple factors. Admittedly, the underlying possibility of prevention is quite variable across all the different events encompassed within this definition: ranging from the simply unexpected readmission to readmissions due to obvious errors. Despite this variance, this definition matches the focus of current reform efforts and research. Furthermore, this definition specifically excludes all index admissions, planned, or elective occurrences.

**Figure 1:**
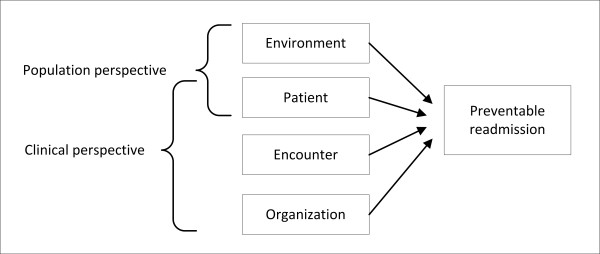
**Conceptual model of the determinants of preventable readmissions**.

An adaptation of an existing health services research framework [19] helps organize and evaluate those factors reported in the literature as influencing preventable readmissions. Under this view, healthcare is the intersection of population health and medical care: the population perspective suggests outcomes are derived in part from individual characteristics as well as the qualities of their environment, whereas the clinical perspective adds the roles of the processes and structure of healthcare encounters. We use these perspectives to consider the preventable readmission determinants as operating within four levels (Figure 1). Patient characteristics include demographics, socioeconomic standing, behaviors, and disease states. The encounter level includes all activities and events associated with the delivery of care for the index hospitalization. The features of the organization that are not specific to a single encounter, but applicable to all encounters in the facility compose the organizational level. Finally, all factors external to the individual and the provider are included in the environmental level. In addition, we recognize this is a simplification of the preventable readmission phenomenon, second order determinants and interactions undoubtedly exist, but the complexity of those relationships is beyond the scope of this review.

### Review methods

**Figure 2:**
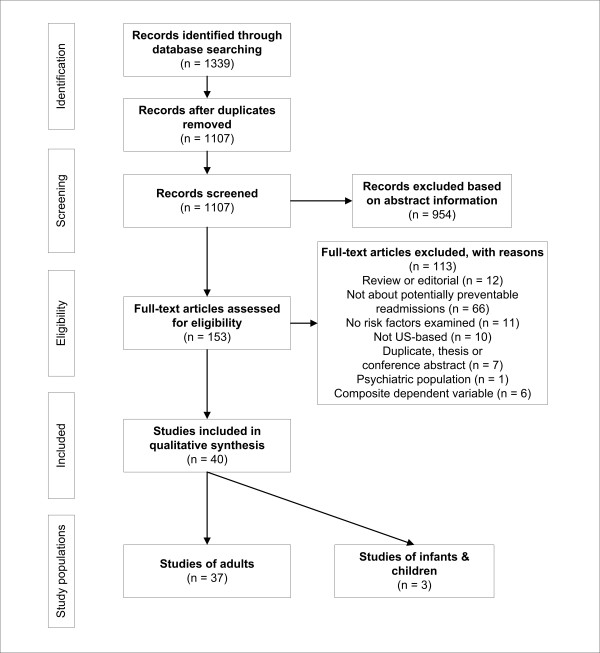
**Search strategy, exclusion and inclusion criteria**.

We undertook a systematic review to identify the factors associated with preventable readmissions following the suggested form of the Preferred Reporting Items for Systematic Reviews and Meta-Analyses (PRISMA) [20]. The search strategy is summarized in Figure 2.

### Information sources and searching

We conducted a review of the English language medicine, health, and health services research literature for research studies dealing with unplanned, avoidable, preventable, or early readmissions. Each of these modifying terms was included in keyword searches of hospital readmission or readmission in Medline, ISI, CINAHL, The Cochrane Library, ProQuest Health Management, and PAIS International. Searches were limited to 2000 to 2009 because the major review by Benbassat and Taragin [3] covered the previous decade. Furthermore, we opted to limit our investigation to the English-language, US healthcare-based literature for the following reasons: while we anticipated patient-level or encounter characteristics would be consistent among other countries, the healthcare environments and organizational vary substantially from the US; and underlying our interest are the relationships of preventable readmissions to US healthcare policy and payment structures. A detailed search strategy is included as Appendix 1. Initial search results yielded 1,107 unduplicated records.

### Study selection

Based on abstract information, we excluded from the initial search set: non-US based studies, studies of psychiatric patients or hospitals, editorials, practice guidelines, reviews, or instances where no indication existed the study was about preventable readmissions. Four members of the research team independently reviewed each record and then arrived at the excluded set through consensus. Our primary search and screening resulted in 153 articles for full text review.

The same four members of the research team independently read the full text of each article and determined its inclusion status. Differences were resolved by consensus after a joint reading session. Articles were retained for inclusion in the review if they meet the following criteria: distinguished between all readmissions and those that were unplanned, early, avoidable, or preventable; investigated potential risk factors or determinants of preventable readmission; and did not combine other outcomes (like mortality or emergency department admissions) with readmissions into composite outcomes. In addition, we reassessed each article according to our previous exclusion criteria. We did not restrict inclusion according to study design. A total of 40 articles met the inclusion criteria after full text review.

Of the 40 articles, three were studies of infant hospitalizations. At this point we determined to exclude these three articles from the review for the following reasons: because infant hospitalizations and surgical procedures are qualitatively different than adult admissions, we thought it would be difficult to combine the two populations in order to make general conclusions or that any contrasts might be artificial; the opportunity to identify patient behaviors and characteristics for intervention is markedly different for infants and children who are totally dependent on others for healthcare decisions; our strategy found so few studies of infants we believed there was not sufficient material for analysis; and, given the limited number, we were concerned our search strategy was biased against finding infant hospitalization studies (we did not specifically include terms that may have found more infant based studies). Therefore, we opted to exclude studies of children and infants. Our final review included 37 studies, all among adult populations.

### Data collection

From each included article, we abstracted the study design, population, setting, type of readmission identified by the authors (unplanned, early, potentially preventable, *et al*.), index condition, the operationalization of readmission (timeframe and cause), and identified risk factors by level. In addition, we noted any models or reasoning that tied the index condition to the readmission, methods to guard against lost to follow-up or selection bias, and statistical methods.

### Assessment

As a means of summarizing the quality of the article and the potential for bias in examining preventable readmissions, we assessed each article according to the presence or absence of three criteria covering the areas of conceptualization, patient linkage, and analysis. Under conceptualization, we looked for studies that explicitly provided a biological, medical, or theoretical model or reasoning tying the index condition to the readmission condition. The presence of such a model, which obviously could take different forms, strengthened the assumption of an underlying probability of preventability of the readmitting condition. While readmissions for the same condition were considered as fulfilling this criterion, *post-hoc *reasoning of results or implicit assumptions of relationships did not. Second, a significant concern in any readmission study is the potential for patients' subsequent admissions to be with another facility. We considered studies that detailed a method to guard against attrition or selection bias as possessing an adequate patient linkage strategy to address these concerns. We looked for the reported strategies to follow or contact patients post-discharge, or the use of shared statewide databases. Finally, we noted articles that made use of multivariate statistics to control for potential confounding factors. Absence of any of these three features represents a potential bias.

## Results

### Study characteristics and risk of bias

A total of 37 studies describe the factors associated with non-psychiatric related readmissions, among adults, defined by the authors as potentially preventable, early, unplanned, or avoidable, to a US hospital after discharge. Retrospective cohorts were the dominate research design [2,5,21-43], followed by prospective cohorts [44-49], case control studies [50-52], and finally case series [53-55]. Through the use of the existing datasets from Medicare [22,32,40], the Health Cost of Utilization Project (HCUP) [2,31], the Veterans' Administration [5], state-specific discharge files [23,25-27,35,41,43], or other secondary sources [30,39], select studies were able to assemble very large sample sizes and include multistate [2,30,31,49] or nationwide coverage [5,22,32,40]. Institution-based studies tended to rely on data abstracted from their own medical records (including electronic sources) [21,24,28,29,34,37,42,47,50-52,54,55], occasionally supplemented with interview data [33,36,38,44-46,48,53].

According to our assessment strategy, the potential for bias is mixed. Nine of the studies meet all three of our quality criteria [22,23,25-27,31,34,45,47]. However, the same number of studies possessed only one or none of the desired characteristics [24,33,37,39,50,52-55]. While the most frequently absent criterion was an explicit conceptual linkage between the index and readmitting condition, most studies meet this requirement by simply limiting the reason for readmission to the same or related diagnosis during the index admission [21,23-29,31,34-36,42,47,49-51]. A handful of studies were able to considered more disparate readmission reasons as preventable by applying accepted definitions of preventable conditions [2,25,43], specifying the phenomena driving readmission [44,45], detailing a clinical link [26], or outlining a full conceptual model [22].

Inadequate designs or methodologies to ensure linkage of the patient's index admission to subsequent readmissions over time and across locations occurred in only 10 studies [21,24,28,29,39,42,50-52,55]. These tended to be single site, or narrowly defined geographical area studies. The single site and smaller studies that meet this criterion reported the use of post-discharge interviews, contacts with family, telephone calls, or physician interviews to improve patient tracking [30,33,36,38,44-46,48]. The use of already linked, shared statewide inpatient databases or large nationwide files such as Medicare helps alleviate concerns that subsequent admissions may have been lost to follow-up.

Confounding and statistical conclusion validity were likely problems in a significant percentage of the studies. In terms of confounding, 14 of the 37 included studies did not analyze their data with multivariate methods [2,24,33,35-37,43,44,49,50,52-55]. Even among those that did use multivariate methods, not all modeling choices meet the necessary statistical assumptions [5,27,46]. However, several studies either utilized methods appropriate to the clustered nature of the hospital discharges [23], or analyzes stratified by organization [26,35].

Finally, although generalizablity was not one of our formal assessment criteria, it bears mentioning. Due to our selection criteria, none of these studies are generalizable to children. In addition, several studies were of very restricted age ranges [41,45,53,55], with those using Medicare data as the most obvious [5,22,32,40]. The restricted age ranges of the Medicare-based studies limits the generalizablity of results, even though these studies had nationwide populations. Also in terms of geography, not all states were represented and more than one state's databases or population were examined on multiple occasions (*e.g*., New York [2,27,31,35,43], California [25,31,39], and Pennsylvania [2,23,41]).

### How has the existing literature defined preventable hospitalizations?

Table 1 summarizes the operationalization of preventable readmission definitions in the literature grouped by the term employed by the authors. As evident, variation triumphs over consistency. For example, among the 16 studies that purported to study early readmissions, there are 15 different combinations of index conditions, readmitting conditions, and timeframes. Although 30 days post-discharge was the most popular choice of time until readmission, it is only one of 16 different timeframes examined and the reason for the selected timeframe was often not provided. Terms frequently are used in combination or as synonyms and different terms are used to describe similar relationships between index and readmitting conditions. For example, two studies described readmitting conditions that can be reasonably assumed to be related to the index admission as potentially preventable [26,31]. At the same time, several studies also examined readmissions for the same condition or complications, but called them early readmissions [21,23,27-29,47,50] or unplanned readmissions [24,34], or unplanned related readmissions [36]. Further complicating matters, seven additional studies also used the term early readmission, but did not provide any strong link between the index and readmission [30,37,38,40,46,48,55].

**Table 1:** Variation terms, definitions, and timeframes in preventable readmission research

Term	Index condition	Readmission condition	Timeframe
Early	Acutely decompensated heart failure	Heart failure or other cardiac cause	90 days[47]

Early	Any condition	Any condition	30 days[30,55]

Early	Any condition	Any condition	41 days[44]

Early	Any condition	Any nonelective readmission	60 days[22]

Early	CABG	Likely to be complications of CABG surgery	30 days[27]

Early	CABG surgery	Any condition	30 days[48]

Early	CHF	CHF exacerbation admission	30 days[50]

Early	CHF	CHF	180 days[21]

Early	Elective laparoscopic colon and rectal surgery	Any condition	30 days[37]

Early	Heart failure	Heart failure	30 days[28]

Early	Heart failure and shock	Any condition or heart failure	30 days[29]

Early	Ileal pouch-anal anastomosis surgery	Any emergent or elective, unplanned readmission	30 days[38]

Early	Multiple chronic illnesses	Any condition	3 to 4 months[45]

Early	Pancreatic resection	Any condition	30 days and 1 year[40]

Early	Pulmonary embolism	Any condition and complications of pulmonary embolism	30 days[23]

Early unplanned	Cardiac surgery	Any condition	30 days[46]

Late unplanned	Pneumonia	Pneumonia	30 days to 1 year[51]

Non-elective and unplanned	Congestive heart failure	Same DRG as index admission	30 days[35]

Potentially avoidable	AMI	AMI - related admissions	56 days to 3 years[25]

Potentially preventable	1^0 ^diagnosis of diabetes or 2^0 ^diabetes diagnosis among high risk conditions	Diabetes - related	30 and 180 days[31]

Potentially preventable	AHRQ's prevention quality indicators	AHRQ's prevention quality indicators	6 months[2]

Potentially preventable	Any condition	Clinically related to index admission	7, 15 and 30 days[26]

Readmissions due to early infection	Surgery	Infection	14 to 28 days[42]

Shortly after discharge	Heart failure	Any condition	30 days[32]

Short-term	Any surgical procedure	Venous thrombo-embolism (AHRQ PSI)	30 days[43]

Unexpected early	Intestinal operations	Any condition (excluding planned)	30 days[33]

Unplanned	Abdominal or perineal colon resection	Related to the primary surgical procedure	90 days[24]

Unplanned	Any acute, short-stay admission	Any unexpected admission	30 days[5]

Unplanned	Any condition	Any condition	Up to 39 days[54]

Unplanned	Any condition	Any condition	31 days[53]

Unplanned	Any non-maternal, substance abuse or against medical advice discharge	Emergent or urgent admissions	30 days[39]

Unplanned	Cancer	Any unplanned	7 days[52]

Unplanned	Cardiac surgery	Related to complications of cardiac surgery	30 days and 6 months[34]

Unplanned related	Ileal pouch-anal anastomosis surgery	Admission resulted from a complication	30 days[36]

Unplanned, non-elective	Traumatic brain injury	Any non-elective or unplanned reason	1 and 5 years[49]

Unplanned, undesirable readmissions	Diabetes	Any non-elective	30 days[41]

However, a few studies provided a careful explanation or justification for relating choice of terminology, index conditions, and readmitting condition. While being thorough, they also used different approaches. For example, Goldfield *et al. *[26] identified five clinically relevant criteria to establish clinically related readmissions: same condition, clinical plausible decompensation, plausibly related to care during index, readmission for a surgical procedure related to index condition, or readmission for surgical procedure for a complication from index. This approach is notable: because it is based on all patient-refined, diagnosis-related groups (APR DRGs) and secondary discharge data, it could be applied by individual hospitals. Also using secondary data, Garcia *et al. *[25] defined potentially avoidable rehospitalizations for acute myocardial infarction (AMI) based on published ambulatory care-sensitive condition definitions. This approach draws on a large literature-base legitimizing the asserted preventability of these admissions. As an example of different approach, in a small clinical study of cardiac surgery patients, Kumbhani *et al. *[34] provided the fairly straightforward and defensible definition for unplanned readmissions as complications resulting from surgery. However, this definition and others like it are more difficult to apply again in other settings, because they rely on clinical judgment and not a reported list of specific diagnostic codes. That is not to say the judgments were incorrect or any less valid, just more difficult to replicate.

### What factors in the literature are associated with preventable patient readmissions?

Given the inconsistent application of terminology, we did not attempt to stratify results by terminology or timeframe for readmission (*i.e*., early, unplanned, preventable, *et al*.). However, because the etiology of readmissions may vary by index condition or procedure, we stratified the index and readmission conditions into four groups for convenience: any or non-condition specific readmissions, cardiovascular-related, other surgical procedures, and all other conditions.

### Any or non-condition specific readmissions

Nine studies [5,22,30,39,44,45,53-55] included index admissions for any cause followed by any cause readmission. In addition, two studies [2,26] defined multiple index and readmitting conditions, but did not stratify analyses by condition thereby presenting overall summary measures of association. The studies are summarized in Table 2. All of these studies predominately examined patient-level factors, and the primary predictor or possible risk factor for preventable readmission is simply general ill health. This theme appears whether formally measured on the Charlson [30,44] or Elixhauser scales [5], reported as worsening of index conditions [53,54], poor self-rated health [44], unmet functional needs [22], or just by the presence of significant chronic conditions [39,55]. Potentially measuring the same underlying patient status, more than one study identified an association between frequent or increased use of the healthcare system and preventable readmission [5,30,44] as well as increasing or elderly age [5,26,53]. In addition, Arbaje *et al. *[22] reported patients who lived alone, or who lacked self-management skills were at risk for early readmission.

**Table 2:** Studies of preventable readmissions with any cause index admission followed by any cause readmission among adults, United States, 2000-2009

Citation	Reported readmission type (and explanation if provided)	Index condition*	Readmit condition	Timeframe	Population and Setting	Design and Sample size	Data source(s)	Risk factors/associated factors	**Conceptually linked admissions**^**†**^	**Strategy for patient linkage**^**‡**^	**Used multivariate statistics**^**§**^
Anderson, Clarke et al [53]	Unplanned	Any condition	Any condition	31 days	Home health patients ≥65 years at home health agency in IL	Case series and qualitative (76)	Chart review, Interviews	*Patient* Elderly** Female** Development of new condition** Worsening of discharge condition** Respiratory conditions** Cardiac conditions** Gastrointestinal** Neurologic symptoms**	No	Yes	No

Anderson, Tyler et al [54]	Unplanned	Any condition	Any condition	Up to 39 days	Transitional care unit patients after ≥3 day acute care stay at transitional care unit in IL	Case series (68)	Chart review	*Patient* Circulatory disorders** Respiratory disorders** Worsening of conditions** Multiple diagnoses**	No	Yes	No

Arbaje et al [22]	Early	Any condition	Any nonelective readmission	60 days	Medicare patients nationwide	Retrospective cohort (1,351)	Medicare Beneficiary Survey, Medicare claim files	*Patient* Living alone Lack self-management skills Unmet functional need No high school diploma *Encounter* Increasing length of stay	Yes	Yes	Yes

Friedman et al [2]	Potentially preventable (preventable in most cases by ambulatory care of standard quality in the several weeks or months prior to admission)	AHRQ's prevention quality indicators	AHRQ's prevention quality indicators	6 months	All patients in the Healthcare Cost and Utilization Project from NY, TN, PA, WI	Retrospective cohort (345,651)	Hospital discharge data, Healthcare Cost and Utilization Project	*Patient* African American Hispanic *Encounter* Medicaid Self-payer	Yes	Yes	No

Goldfield et al [26]	Potentially preventable (which types of admissions were at risk of generating a readmission)	Any condition	Clinically related to index admission	7, 15 and 30 days	All inpatient encounters in FL	Retrospective cohort (242,991)	Hospital discharge data	*Patient* Age greater than 75 years old *Organizational* Hospital	Yes	Yes	Yes

Hasan et al [30]	Early	Any condition	Any condition	30 days	≥18 years and admitted by hospitalist or internist in six academic medical centers	Retrospective cohort (10,946)	Interviews from multicenter trial, Hospital databases	*Patient* Married Has regular physician Increasing Charlson index Increasing admission in last year *Encounter* Medicaid Medicare Self-pay Length of stay >2 days	No	Yes	Yes

Novotny and Anderson [44]	Early	Any condition	Any condition	41 days	English speaking patients ≥18 years from single IL medical center	Prospective cohort (1,077)	Interviews, Hospital databases	*Patient* Diabetes Increasing number of doctor visits in past year Increasing number of hospitalizations in past year Poor self-rated health status Increasing Charlson score Unemployed Depression Heart failure Marital status *Encounter* Increasing length of stay Medicare/Medicaid Discharge to home healthcare Discharge to healthcare facility	Yes	Yes	No

Parker et al [39]	Unplanned	Any non-maternal, substance abuse or against medical advice discharge	Emergent or urgent admissions	30 days	Kaiser Permanente pharmaceutical patients from multiple CA hospitals	Retrospective cohort (6,721)	Existing study database	*Patient* COPD Diabetes Diabetes with complications Paraplegia Metastatic solid tumor	No	No	Yes

Schwarz [45]	Early	Multiple chronic illnesses	Any condition	3 to 4 months	Patients ≥65 years and functionally impaired in 2 ADL from two hospitals	Prospective cohort (60)	Chart review, Interviews	*Environment* Social support negatively associated with readmission	Yes	Yes	Yes

Timms et al [55]	Early	Any condition	Any condition	30 days	Patients ≥65 years from single SC hospital	Case series (127)	Chart review	*Patient* Female** Heart disease**	No	No	No

Weeks et al [5]	Unplanned	Any acute, short-stay admission	Any unexpected admission	30 days	VA enrollees ≥65 years nationwide	Retrospective cohort (3,513,912)	VA/Medicare combined dataset	*Patient* Increasing age Male Increasing comorbidity (Elixhauser score) Index admission as a readmission (history of readmits) *Encounter* Increasing length of stay *Organizational* Index admission to VA hospital *Environment* Rural	No	Yes	Yes^||^

* All exclusion criteria or specific diagnostic codes not reported - see original article for additional details.

** Study did not compare readmissions with non-readmissions so factors are from descriptive statistics/reports only

^† ^Explicitly specified a biological, theoretical or conceptual model linking the readmission condition to the index condition (includes readmissions for same condition)

^‡ ^Specified a strategy or research design to guard against loss to follow up

^§ ^Used multivariate statistics

^||^Modeling technique did not account of non-independence of observations in analysis

AHRQ = Agency for Healthcare Research and Quality

VA = Veterans' Affairs

ADLs = Activities of daily living

Studies of any cause index admission and readmissions limited examination of the encounter level to a few general factors. Four studies reported an association between increasing length of stay during the index hospitalization and readmission [5,22,30,44]. Also, patients who were covered by Medicare [30,44], Medicaid [2,30,44], or who were self-payers [2,30] were reportedly more likely for readmission than those with private insurance. Finally, in a univariate analysis, Novonty and Anderson [44] reported discharge to home healthcare or to another healthcare facility were associated with early readmissions.

The organizational and environmental levels received even less attention. Weeks *et al*.' [5] study of urban and rural veterans was the only study in the entire review to consider patient, encounter, organizational, and environmental level factors. In terms of the environment, they reported rural veterans had higher odds of unplanned readmissions. For the organizational level, they also reported if the site of index admission was a VA hospital, the odds of readmission were higher. However, the modeling approach didn't account for within-site clustering. Although through a different approach, Goldfield *et al. *[26] also demonstrated that at an overall level, some characteristic of the index hospital matters, as readmission rates varied greatly between facilities. Finally, the research by Schwarz [45] suggests a possible intervention for patients in need of assistance. In her study, patients' with higher levels of social support were less likely to be readmitted early.

### Cardiovascular-related index admissions and readmissions

Thirteen studies considered readmission where the index condition was AMI [25], heart failure [21,28,29,32,35,47,50], coronary artery bypass graft (CABG) surgery [27,48], cardiac surgery [34,46], or pulmonary embolism [23]. (See Table 3.) On patient characteristics, the above studies were consistent on the increased risk of early, unplanned, or avoidable readmissions for patients with: existing heart disease [25,27,32], diabetes [27,32,46,48], COPD [27,29,46], renal dysfunction/failure [32,46], other complex co-morbid conditions [27,32], and higher patient severity scores [23,34]. In terms of gender, women were more likely to be readmitted early for a cardiac-related cause after acutely decompensated heart failure [47], or for complications related to CABG surgery [27], or for any unplanned reason after cardiac surgery [46]. In contrast, Harja *et al. *[29] reported among heart failure and shock patients, men were more likely to be readmitted early with a diagnosis of heart failure than women. Only Hannan *et al. *[27] reported increasing age was associated with readmission and only Aujesky *et al. *[23] found African American patients were more likely than White patients to be readmitted early after pulmonary embolism.

**Table 3:** Studies of preventable readmissions of cardiovascular-related index admissions and readmissions among adults, United States, 2000-2009

Citation	Reported readmission type (and explanation if provided)	Index condition*	Readmit condition	Timeframe	Population and Setting	Design and Sample size	Data source(s)	Risk factors/associated factors	**Conceptually linked admissions**^**†**^	**Strategy for patient linkage**^**‡**^	**Used multivariate statistics**^**§**^
Ahmed et al [21]	Early	Congestive heart failure primary discharge diagnosis	Congestive heart failure	180 days	Congestive heart failure patients from VA medical center in TX	Retrospective cohort (198)	Hospital databases	*Patient* Decreasing temperature	Yes	No	Yes

Aujeskey et al [23]	Early	Pulmonary embolism	Any and complications of pulmonary embolism (recurrent venous thrombo-embolism and bleeding)	30 days	Patients ≥18 years in PA	Retrospective cohort (14,426)	Pennsylvania Healthcare Cost Containment Council database	*Patient* African American (any or venous thromboembolism) Increasing PESI risk class (any cause only) *Encounter* Medicaid Discharge to home with supplementary care (any cause) Left hospital against medical advice (any cause only) *Organizational* Hospital teaching status (bleeding only) Non-Pittsburg area	Yes	Yes	Yes

Ferraris et al [46]	Early unplanned	Cardiac surgery	Any condition	30 days	Cardiac patients from single WV medical center	Prospective cohort (2,650)	Hospital database, Interviews	*Patient* Female Diabetes Preoperative atrial fibrillation COPD Renal dysfunction *Environment* Residential zip code	No	Yes	Yes^||^

García et al [25]	Potentially avoidable	Acute myocardial infarction	Acute myocardial infarction - related admissions	56 days to 3 years	Coronary artery disease in CA	Retrospective cohort (683)	California Hospital Outcomes Validation Project dataset	*Patient* AMI history *Encounter* Medicaid Less likely with CABG on admission	Yes	Yes	Yes

Hallerbach et al [50]	Early	Congestive heart failure	Congestive heart failure exacerbation admission	30 days	Congestive heart failure patients from single PA hospital	Case control (58)	Chart review	No statistically significant factors reported	Yes	No	No

Hannan et al [27]	Early	Coronary artery bypass graft	Likely to be complications of Coronary artery bypass graft surgery	30 days	Coronary artery bypass graft surgery patients in NY	Retrospective cohort (16,325)	New York State's Cardiac Surgery Reporting System linked with the Statewide Planning and Research Cooperative System	*Patient* Increasing age Women Body surface area Myocardial infarction 7 days prior Femoral disease Congestive heart failure Chronic obstructive pulmonary disease Diabetes Hepatic failure Dialysis *Encounter* Low annual surgeon volume Discharge to skilled nursing or rehabilitation facility Increasing length of stay *Organizational* High hospital risk adjusted mortality rate	Yes	Yes	Yes^||^

Harjai, Nunez et al [28]	Early	Heart failure	Heart failure	30 days	Heart failure patients from single LA hospital	Retrospective cohort (576)	Hospital databases, Chart review	*Encounter* Treatment with angiotensin-converting enzyme and aspirin	Yes	No	Yes

Harjai, Thompson et al [29]	Early	Heart failure and shock	Any condition or heart failure	30 days	Heart failure and shock patients from single LA hospital	Retrospective cohort (434)	Hospital databases	*Patient* COPD (any cause and HF) No. of hospitalizations in prior 6 months (any cause and HF) Male (HF only) Increasing blood urea nitrogen (any cause only)	Yes	No	Yes

Howie-Esquivel and Dracup [47]	Early	Acutely decompensated heart failure	Primary diagnosis of heart failure or other cardiac cause	90 days	Heart failure patients from single CA academic medical center	Prospective cohort (44)	Chart review	*Patient* Female *Encounter* Increasing length of stay	Yes	Yes	Yes

Keenan et al [32]	Readmissions to the hospital shortly after discharge	Heart failure	Any condition	30 days	Fee for service Medicare Parts A and B nationwide	Retrospective cohort (1,129,210)	Medicare inpatient, outpatient, and carrier Standard Analytic Files, Medicare Enrollment Database, National Heart Failure Project database	*Patient* History of coronary artery bypass graft surgery less likely Congestive heart failure Acute coronary syndrome Arrhythmias Cardiorespiratory failure and shock Valvular and rheumatic heart disease Vascular or circulatory disease Chronic atherosclerosis Other heart disease Paralysis Stroke Renal failure COPD Diabetes Fluid disorders Urinary tract infections Gastrointestinal disorders Severe hematologic disorder Nephritis Cancer Liver disease Asthma Pneumonia Drug/alcohol abuse or psychosis Fibrosis of the lung Protein-calorie malnutrition (validation dataset not reported)	No	Yes	Yes

Kumbhani et al [34]	Unplanned	Cardiac surgery	Related to complications of cardiac surgery	30 days and 6 months	Underwent intra- operative online monitoring of myocardial tissue pH at VA medical center in MA	Retrospective cohort (221)	Hospital databases	*Patient* Low pH at end of bypass Postoperative atrial fibrillation High ASA class Preoperative ejection fraction *Encounter* Length of stay less than 6 days Myocardial tissue pH < 6.85 at the end of bypass	Yes	Yes	Yes

Lagoe et al [35]	Non-elective and unplanned	Congestive heart failure	Same DRG as index admission	30 days	Congestive heart failure patients from multiple sites in Syracuse	Retrospective cohort (Not reported)	New York Statewide Planning and Research Cooperative System	*Organizational* Rates varied by hospital	Yes	Yes	No

Sun et al [48]	Early	CABG surgery	Any condition	30 days	Low risk CABG patients from Single DC hospital	Prospective cohort (2,157)	Hospital databases, Interviews	*Patient* Diabetes	No	Yes	Yes

* All exclusion criteria or specific diagnostic codes not reported - see original article for additional details.

^† ^Explicitly specified a biological, theoretical or conceptual model linking the readmission condition to the index condition (includes readmissions for same condition)

^‡ ^Specified a strategy or research design to guard against loss to follow up

^§ ^Used multivariate statistics

^||^Modeling technique did not account of non-independence of observations in analysis

Both the risk of potentially avoidable AMI-related readmissions [25], and early readmission for after pulmonary embolism [23] were higher for Medicaid enrollees. The risk of early readmission was higher for patients discharged home with supplementary care [23], to skilled nursing or rehabilitation facility [27], or who left hospital against medical advice [23]. Increasing length of stay was a risk factor for early heart disease readmissions after acutely decompensated heart failure [47] and for 30-day readmits for CABG complications [27]. However, Kumbhani *et al. *[34] recently reported unplanned readmission related to complications of cardiac surgery were more likely for patients with a length of stay fewer than six days. While these general findings are similar to the studies of any cause readmissions, the studies of the cardiovascular-related group were able to go into more detail. For example, García *et al. *[25] report the risk of AMI-related readmissions decreases when CABGs were performed on admission, and Kumbhani *et al. *[34] found a myocardial tissue pH < 6.85 at the end of the bypass increased the odds of 30-day readmission more than six-fold. Finally and particularly noteworthy, is the increased risk of early readmission due to complications of CABG surgery when the procedure was performed by a surgeon with low annual CABG volumes reported by Hannan *et al. *[27]. This was the only study to examine a characteristic of the individual provider associated with the index admission.

Again, organizational and environmental level factors were explored infrequently. As a global measure, Lagoe *et al. *[35] found unplanned readmissions for congestive heart failure varied by hospital in Syracuse, NY. This tends to suggest organizational characteristics matter in cardiovascular-related preventable readmissions, but care must be taken in interpreting organizational level findings as no risk or case mix adjustment was reported. In support of this conclusion, Keenan *et al. *[32] employed among the most sophisticated modeling techniques in the review to account for clustering and different patient mixes. However, because they did not examine any organizational level factors, the reported variance in the hospital specific intercepts again only suggests some organization factors are at play. More specific factors were examined by Aujesky *et al. *[23], who found 30-day readmissions were higher for teaching hospitals and for hospitals located in particular parts of the state. While these authors did not specifically control for case mix, they did conduct site-specific analyses to look for specific variation in their models. Additionally, in their study of readmissions due to complications of CABG surgery, Hannan *et al. *[27] modeled the higher level determinants like hospital risk adjusted mortality rates, but the study relied on ordinary logistic regression violating independence assumptions. At the environmental level, Ferraris *et al. *[46] reported the patient's zip code was associated with unplanned readmissions. However, because the authors used ordinary logistic regression, the statistical significance may be solely due to underestimated standard errors.

### Surgical procedures

Table 4 summarizes five studies that examined preventable readmissions after colorectal or lower intestinal surgeries [24,33,36-38], the two after any type of surgical procedure [42,43] and one study on pancreatic surgery among cancer patients [40]. Results for this group are a little sparse, however, as three employed only univariate statistics [33,37,43] and two found no statistically significant factors [24,36]. Still, a few factors are repeatedly identified within this group. Again, patient co-morbidity was associated with preventable readmissions after ileal pouch-anal anastomosis [38] and pancreatic resection surgeries [40]. Also, for both pancreatic cancer [40] and colorectal surgery patients [33], those readmitted appear to have longer inpatient stays than those who are never readmitted.

**Table 4:** Studies of preventable readmissions related to surgical procedures among adults, United States, 2000-2009

Citation	Reported readmission type (and explanation if provided)	Index condition*	Readmit condition	Timeframe	Population and Setting	Design and Sample size	Data source(s)	Risk factors/associated factors	**Conceptually linked admissions**^**†**^	Strategy for patient linkage^‡^	**Used multivariate statistics**^**§**^
Azimuddin et al [24]	Unplanned	Abdominal or perineal colon resection surgery	Related to the primary surgical procedure	90 days	Colorectal surgery patients from single PA hospital	Retrospective cohort (249)	Chart review	No statistically significant factors found	Yes	No	No

Kiran et al [33]	Unexpected early	Intestinal operations	Any condition (excluding planned)	30 days	Colorectal surgery service patients single OH hospital	Retrospective cohort (553)	Chart review, Interviews	*Encounter* Increasing length of stay	No	Yes	No

Medress and Fleshner [36]	Unplanned related (a direct consequence of the recent operation)	Ileal pouch-anal anastomosis surgery	Admission resulted from a complication	30 days	Inflammatory bowel disease patients requiring colectomy from single CA hospital	Retrospective cohort (202)	Hospital databases, Interviews	No statistically significant factors found	Yes	Yes	No

O'Brien [37]	Early	Elective laparoscopic colon and rectal surgery	Any condition	30 days	Colorectal surgery patients from single OH hospital	Retrospective cohort (820)	Hospital databases	*Patient* Pulmonary disease Inflammatory bowel disease *Encounter* Perioperative steroids Conversion from laparoscopic to open operation	No	Yes	No

Ozturk et al [38]	Early	Ileal pouch-anal anastomosis surgery	Any emergent or elective, unplanned readmission	30 days	Ileal pouch-anal anastomosis surgery patients from single OH hospital	Retrospective cohort (3,410)	Hospital database, Interviews	*Patient* Comorbidity *Encounter* Laparoscopic approach Synchronous protocolectomy Postoperative blood transfusion	No	Yes	Yes

Reddy et al [40]	Early	Pancreatic resection	Any condition	30 days and 1 year	Pancreatic cancer patients, ≥66 years in SEER and Medicare Parts A and B nationwide	Retrospective cohort (1,730)	SEER-Medicare Linked Data	*Patient* Increasing Charlson score (1 year) *Encounter* Increasing length of stay (30 day and 1 year) Distal pancreatectomy (30 day)	No	Yes	Yes

Scott et al [42]	Readmissions due to early infection	Surgery	Infection	14 to 28 days	Received prophylactic antibiotic prior to surgery from single NY hospital	Retrospective cohort (9,016)	Hospital databases	*Encounter* Skin or tissue biopsy Dialysis shunt Endarterectomy Non-cardiac vascular repair Early infection	Yes	No	Yes

Weller et al [43]	Short-term	Any surgical procedure	Venous thrombo-embolism (AHRQ PSI)	30 days	Surgical patients from NY	Retrospective cohort (4,906)	New York Statewide Planning and Research Cooperative System	*Patient* Female** White non-Hispanic** Increasing age**	Yes	Yes	No

* All exclusion criteria or specific diagnostic codes not reported - see original article for additional details.

** Study did not compare readmissions with non-readmissions so factors are from descriptive statistics/reports only

^† ^Explicitly specified a biological, theoretical or conceptual model linking the readmission condition to the index condition (includes readmissions for same condition)

^‡ ^Specified a strategy or research design to guard against loss to follow up

^§ ^Used multivariate statistics

AHRQ = Agency for Healthcare Research and Quality

SEER = Surveillance, Epidemiology and End Results

PSI = Patient safety indicators

As would be expected, because they focused on surgical procedures, the studies in this group indentified several unique possible risk factors occurring during the index encounter. Among colorectal surgery patients, readmissions were more common among patients after conversion from laparoscopic to open operation or perioperative administration of steroids [37]. The odds of early readmission after ileal pouch-anal anastomosis were higher for laparoscopic approach, synchronous protocolectomy, or postoperative blood transfusion [38]. Finally, Scott *et al. *[42] reported numerous factors associated with early readmissions due to infections.

### Other conditions

The final five studies, displayed in Table 5, cover the diverse index conditions of diabetes [31,41], pneumonia [51], traumatic brain injury [49], and cancer [52]. Among diabetics, both studies indicated a greater risk of potentially preventable [31] or unplanned [41] readmissions for African Americans, but present conflicting results for Hispanics. Furthermore, in Robbins and Webb's [41] large cohort, they also identified increasing age, severity class, previous utilization, increasing length of stay, and discharge to other institutions or home health as risk factors. El Solh *et al. *[51] examined unplanned pneumonia readmissions among the elderly, and was one of the few studies to include measures of patient dependency. In a similar vein, A small study by Weaver *et al. *[52] concluded that inadequate care giver support was more common among unplanned readmissions. Finally, related to the possibility of support for high-risk patients outside the acute care setting, traumatic brain injury patients who lived in private residences were less likely to be readmitted for non-elective reasons.

**Table 5:** Studies of preventable readmissions for other conditions among adults, United States, 2000-2009

Citation	Reported readmission type (and explanation if provided)	Index condition*	Readmit condition	Timeframe	Population and Setting	Design and Sample size	Data source(s)	Risk factors/associated factors	**Conceptually linked admissions**^**†**^	**Strategy for patient linkage**^**‡**^	**Used multivariate statistics**^**§**^
El Solh et al [51]	Late unplanned	Pneumonia	Pneumonia	30 days to 1 year	Patients ≥65 years from 3 university affiliated hospitals	Case control (408)	Multiple hospital databases	*Patient* Increasing ADL score (more dependent) Smoking Swallowing dysfunction Pneumovax (less likely) Angiotensin-converting enzyme inhibitor (less likely) Tranquilizer	Yes	No	Yes

Jiang et al [31]	Potentially preventable (complication more likely preventable with effective postdischarge care)	1^0 ^diagnosis of diabetes or 2^0 ^diabetes diagnosis among high risk conditions	Diabetes - related	30 and 180 days	Diabetics ≥18 years in Healthcare Cost and Utilization Project from CA, MO, NY, TN, VA	Retrospective cohort (130,751)	Healthcare Cost and Utilization Project	*Patient* Hispanic (30 and 180 days) Black (180 days)	Yes	Yes	Yes

Marwitz et al [49]	Unplanned, non-elective	Traumatic brain injury	Any non-elective or unplanned reason	1 and 5 years	NIDRR Traumatic Brain Injury Program from 17 medical centers nationwide	Prospective cohort (895)	NIDRR Model Systems for Traumatic Brain Injury database	*Environment* Private residence less likely	Yes	Yes	No

Robbins and Webb [41]	Unplanned, undesirable readmissions	Diabetes	Any non-elective	30 days	Diabetics ages 25 - 84 from Philadelphia	Retrospective cohort (291,752)	Pennsylvania Healthcare Cost Containment Council database	*Patient* Male Increasing age less likely African American Hispanic less likely Asian Other/unknown race-ethnicity Increasing severity class Increasing number of prior hospitalizations *Encounter* Medicaid less likely than Medicare Private insurance less likely than Medicare Uninsured/self-pay less likely than Medicare Increasing length of stay Discharged to other institution Discharged to home health Discharged against medical advice	No	Yes	Yes

Weaver et al [52]	Unplanned	Cancer	Any unplanned	7 days	Cancer patients from cancer center in PA	Case control (78)	Chart review	*Patient* Gastrointestinal cancer Financial or insurance problems Living alone *Environment* Caregiver difficulty	No	No	No

* All exclusion criteria or specific diagnostic codes not reported - see original article for additional details.

^† ^Explicitly specified a biological, theoretical or conceptual model linking the readmission condition to the index condition (includes readmissions for same condition)

^‡ ^Specified a strategy or research design to guard against loss to follow up

^§ ^Used multivariate statistics

NIDRR = National Institute on Disability and Rehabilitation Research

## Discussion

The current literature on preventable readmissions in the US contains evidence from a variety of populations, locations, settings, designs, and conditions. If a single common set of consistent patient-level risk factors can be distilled from this review it would include a variety of measures of poor-health or frailty: co-morbidities [5,25,27,30,32,38-40,44,46,55], increasing severity class [23,34,41], increasing age [5,26,27,41,53], general poor health [44,53,54], or high previous utilization of the healthcare system [5,29,30,41,44]. In addition, some studies highlighted racial/ethnic disparities in preventable readmission for diabetics [31,41], patients with pulmonary embolism [23], and other preventable conditions [2]. However, these potential risk factors are common to other investigations of hospitalization. In Jencks *et al. *[1] recent examination of rehospitalizations (where they make no claim to preventability), they identified similar indicators of patient ill-health and disparities by race, socio-economic status, and geography. Other types of healthcare utilization show similar patterns: disparities according to race/ethnicity [56] and risks based on age [57] for hospitalizations due to ambulatory care sensitive hospitalizations, and those with poor health are more likely to be frequent users of emergency departments [58].

The combined results of encounter level factors run along similar lines. Across multiple conditions, encounters covered by Medicaid [2,23,25,30,44] or self-pay [2,30] were indicators of increased odds of subsequent preventable readmissions; these are again probably proxies for either socio-economic status or access to primary care issues. In addition, while length of stay is encounter-specific and identified as an associated factor in multiple studies [5,22,27,30,41,44,47], it may in part reflect underlying patient health [59]. The same may be true for those studies that indicated discharge to some other care facility or supplemental care were associated with readmission [23,27,44].

Intuitively and from a few studies in this review, we know that the admitting hospital may make a difference on subsequent readmissions. We cannot definitively say why or how. We do not know if the admitting hospital actually exerts some effect (through structures, policies, and procedures), or if it is merely variation for which examinations must account. Several studies documented that hospitals are different [23,26,30,32,35], but very few looked for organizational-level factors. Even when organizational factors are explicitly examined, we are still uncertain about the magnitude or validity of the effect because statistical assumptions were violated [5,27].

In similar fashion, the results of factors at the environment level are, on balance, more suggestive than informative at this point. Living in a private residence [49], difficulty in getting care givers [52], or lack of social support [45] are really features of the patient's environment. However, only the study by Schwarz [45] used multivariate statics, theoretically linked the index and readmission, and ensured adequate patient follow-up. Even then, the study focused on a small, narrowly defined population. Ferraris *et al. *[46] found a patient's zip code associated with unplanned readmissions, but knowing what these results means is obscured because we know nothing about the resources or socioeconomics of the areas, and the modeling fails to account for multilevel measurement. By specifically modeling the zip code, Ferraris *et al*. were asserting that the environment has an effect. Likewise, Weeks *et al. *[5] found effects for rural residence. The result is intriguing, but the questions about the underlying mechanism accounting for the risk it raises are more logically answered by features of the environment: is it access to specialists, primary care, or rehabilitation and preventative services? While residence could be considered a patient-level variable, we would argue that rurality is more about the patients' context, and less about their own characteristics and behaviors.

The current research is missing in-depth examinations of more than one aspect of preventable readmissions. While it is fairly clear that patients with markers of general poor health are more likely to come back to the hospital, our knowledge about encounter-level factors is predominately related to length of stay and payer. Variance in the former depends substantially upon condition, and the latter is confounded by socioeconomic status, access, and a host of other factors. Few studies ventured to examine organizational and environmental factors. Fortunately, these gaps can be readily addressed. All multi-facility investigations using large databases could easily incorporate organizational level factors and utilize random effects or other cluster adjustments. The now more widespread appreciation of statistical methods for handling clustered data and improved computer power means the more sophisticated statistical methods utilized by a few studies in this review can be replicated. Furthermore, numerous structural and performance measures are available from existing surveys. Additionally, factors measured at the zip code level, like poverty or availability of primary care, are easily attainable and provide information on neighborhood effects and area resources. Again, these factors can be incorporated into models given the appropriate choice of statistical technique.

### Variance in definitions makes drawing on the existing literature difficult

This paper has focused on preventable readmissions, but this is a term of convenience because the underlying possibility of prevention is variable across different readmissions. Unfortunately, it is frequently difficult to decide just how preventable the readmissions truly are due to numerous timeframes, the pervasive lack of conceptual clarity, and the varying use of terminology. Synthesizing results is thus hampered. These definitional difficulties call for a clear, shared vocabulary; for the choice of term makes a difference, as it not only indicates the degree to which the readmission is preventable, but also suggests by what mechanism prevention may be achieved.

This review contributes to that effort as some of the studies reviewed make strong efforts at conceptual and definitional clarity. From those studies, we can start to apply some common definitions and order to these terms. First, the term 'early' stresses the temporal association between the index and subsequent admissions. However, causality is not definite, because both elective and non-elective readmissions can occur shortly after discharge [8]. 'Unplanned or non-elective' readmissions are not scheduled occurrences part of the medical process and undesired returns to the hospital [24,41]. These labels are more descriptive and restrictive than simply 'early' because they eliminate some obviously non-preventable readmissions from consideration. Additionally, the word 'unplanned' sounds more like an aberrant event in the medical intervention initiated at the hospital, which ties the readmission to the care received during the index hospitalization. Finally, two terms clearly indicate a belief that intervention could effectively reduce the probability of readmission and employ more causal-type language. 'Potentially avoidable' draws upon the language of ambulatory care sensitive conditions, signifying appropriate, quality primary care can prevent readmission [25]. By utilizing this established literature base, this label indicates a general strategy to reduce readmissions by improving the quality of, and access to, post-discharge care and patient management. 'Potentially preventable' was used by Goldfield *et al. *[26] to describe clinically related, needless readmissions that quality care, discharge planning, follow up, or improved coordination would avert; this terminology not only claims a high expectation of preventability, but also implies broader opportunities for intervention inside and outside the hospital. Descriptions of readmissions adhering to the above terms and concepts would greatly facilitate comparisons between studies and simplify the national conversation on reform.

### Methodological challenges make applying the existing literature to local practice difficult

Researchers, administrators, and clinicians have over many years pursued identification of readmission cases through predictive models with intentions of effectively intervening to extend or support a patient's care after discharge. While this review identified some consistent factors for such a model, it also catalogued a great deal of variety. For every reasonably consistent factor, like increasing co-morbidity scores, older age, or race/ethnicity disparities, there appeared to be multiple, detailed factors specific to the index and readmitting condition, like type of cardiovascular treatments, intraoperative measurements, surgical approaches, or specific existing conditions. This suggests a statistical model of just preventable readmissions may prove to be too elusive and that we should focus on condition specific preventable readmissions, either through stratified models or categorical dependent variables. While more complicated, that approach may prove more effective. Studies that do not restrict analysis to a single set of clinically-related index and readmitting conditions are most likely limited to effectively modeling only general risk factors, because the distinctive risks for various conditions may be may be lost in, or overpowered by, variables that apply to all conditions. Unfortunately, it is probably the condition-specific risks that provide the most opportunity for effective intervention within the hospital and in post-discharge settings. However, as much of the organizational and environmental factors are yet untapped, more information in the future may allow the question to be reexamined.

Four practical methodological challenges also hinder application of results in local practice. First, the studies in this review included both analyses of secondary linked datasets and those that relied on primary data collection and chart review. There is a difficulty in rectifying these two methods. Because primary data collection allows for many more detailed factors that may not be available in administrative databases, some findings may not be able to be utilized by those working in secondary data. In addition, the large sample sizes of the linked datasets may have indentified factors that will not be detectable in single-site studies. If it takes statewide or nationwide databases to identify statistically significant predictors because their effects are so small, it is difficult to assume any single facility will be able to generate the same level of precision in their own models. This is particularly true if we are going to have to stratify predictive models by specific condition or procedure. Third, the ability to adequately identify patients' previous and subsequent admissions may be very difficult for some facilities. The majority of studies relied on linked databases to ensure that all admissions to other facilities were being captured. Otherwise, extensive primary data collection was required. If facilities opt not to invest in primary data collection and patient follow-up, the ability of any single organization to identify their facility-specific risk factors for preventable readmissions may have to wait for fully developed local heath information exchange to follow patients between providers. Alternatively, the agency responsible for aggregating discharge claims within each state may have to take on the burden of patient matching. Finally, while in this review we have already advocated for more appropriate statistical techniques to account for the clustered nature of readmission, we recognize this type of modeling is not easy. Random-effects modeling requires expertise, specialized software, and sufficient computing power. Some organizations, like academic medical centers or VA facilities with access to health service research postdoctoral fellows, may be better positioned to engage in this type of predictive modeling. For other organizations, these approaches may be beyond their in-house capabilities.

### Strategies for hospitals

If the conventional wisdom is to be believed, the cost of preventable readmissions will be borne principally by hospitals. However, as suggested in the introduction and as the existing literature has borne out, preventable readmissions are influenced by factors at the patient, encounter, organizational, and environmental levels. Which of these factors are actually in the hospitals' control or even amenable to direct influence?

Obviously, individual patient characteristics require significant consideration for those planning any interventions. It is an interesting contradiction that patient-level characteristics were the dominant area of inquiry for the reviewed studies, but most of these characteristics seem to be out of the hospitals' direct control. As O'Brien noted, 'unfortunately, many of these patient characteristics cannot be altered' [[37] p2142]; a somewhat fatalistic comment, suggesting that research will need to increasingly identify behaviors and or contexts that can be targeted by interventions and evaluations. Furthermore, the increased risk for a preventable readmission for patients discharged against medical advice [23,41] does not particularly bode well for any ideas that the hospital will be able to effectively influence subsequent health behaviors or even monitor resource utilization [50]. However, more than one study in the earlier review by Benbassat and Taragin [3] found interventions to provide post-discharge support or assistance reduced readmissions, and more recently, some systems such as Geisinger [60] report success with patient-follow up after discharge.

Several encounter-level risk factors identified in this review, particularly those pertaining to specific procedures and medical interventions, are changeable by hospitals. In fact, the reviewed literature makes a few explicit recommendations, but these changes or improvements to clinical care while in the hospital are very condition-specific [21,34,43,47,50,51]. The fact that there are so few specific recommendations for providers of care is not surprising because much of the literature was admittedly focused primarily on measurement methods [26,30,32,35,39,44] and policy issues broader than intra-hospital operations [5,22,25,27,31]. Therefore, beyond the few clinically-specific recommendations, the bulk of the remaining encounter-level risk factors hospitals either actually cannot change (such as who pays for the encounter or if the patient leaves against medical advice) or a simple, all-encompassing recommendation that is much more difficult (as in the case of length of stay, which is subject to a host of condition-specific clinical and payer influences). Similarly, hospitals may have limited or no effect on the supply or quality of primary care providers or home health, rehabilitation, or skilled nursing programs or facilities that may impact readmissions.

As deterministic actors, hospitals can make changes to their structure and processes and push back against environmental forces. Although hospitals can clearly change themselves and at least try to change the environment of their patients, the existing literature gives little guidance. As noted, the reviewed studies did not identify any organizational-level factors that can be easily targeted for change. Environmental-level determinants were also infrequently examined, but at least there we have some ideas of plausible interventions, mostly in the arena of changing patients' immediate support network. For example, Weaver *et al. *[52] advised coordination with social workers or case managers during the discharge of cancer patients, and Timms *et al. *[55] advocated for more qualitative information gathering through interviews with the patients, family members, and caregivers about the needs of elderly patients. These recommendations can be empirically tested in highly variable settings by multi-hospital systems or independent hospitals working on a joint program of research using quasi-experimental designs.

So what should hospitals do? Multiple options are available, but the choice of approach, in part, reflects the organization's underlying assumptions about the causes of readmissions, the applicability of predictive models, and the forthcoming financial policies. One viewpoint is that preventable readmissions are clearly a measure of overall hospital quality and that all preventable readmissions, regardless of causes, have some underlying driving factors [1-3,5,7-11,26,32,41]. While this view would allow for statistical modeling as an effective means of performance measurement [26,32], philosophically it implies that the search for individual risk factors or single interventions is too narrow in scope. If one accepts that preventable readmissions are failures at multiple processes, levels, and structures of healthcare, then these readmissions stand as a global indicator, not a single data point the organization tries to move; the potential changes in reimbursement are not intended to change a targeted practice or behavior, but to spur overall quality. That viewpoint suggests the solution to preventable readmissions is improvement in overall quality. That is definitely a hospital-centric view, where the efforts of the hospital are paramount in affecting preventable readmissions. In support of this view is that evidence indicates some hospitals are both better than expected and better than their peers in terms preventable readmission rates [26]. Maybe these are the higher quality hospitals, or simply those who care for patients with lower severity conditions, or are located near more higher quality primary care and post-discharge care providers. For those organizations performing poorly on preventable readmissions, the implication is the need for organization-wide transformation. Transformation is not the adoption of a single technology or approach, but a profound change in the entire organization's culture and processes that improves quality [61-64]. Unfortunately, the transformation in healthcare organizations has not been easily or widely achieved [65].

A second general viewpoint is that preventable readmissions are not about the quality of care [33,36]. Preventable readmissions are more about the person receiving care [24,29,44-46,48,55] and the viewpoint is marked by phrases like 'unpredictable sequel' [33] and 'cannot be predicted' [24,36]. While not as dismissive of preventable readmissions as a marker of quality as the preceding quotations, those focusing on patients' post-discharge experiences, contexts, and resources [2,22,25,31,52,66] could also be considered as sharing this extra-hospital viewpoint. This view is in stark contrast to the hospital-centric viewpoint, because whether preventable readmissions occur from pre-existing co-morbidities, health behaviors, or access to primary care, these things are all beyond the scope of services provided by the traditional inpatient setting. Reimbursement reform, therefore becomes an unfair financial penalty [67] that hospitals try to avoid through various targeted initiatives like improved information systems [18], case managers [52], and post-discharges follow up [60]. The underlying theme of these approaches and this extra-hospital view is that patients in some fashion have to be actively managed, because the negative financial outcomes are too great to take a passive role. For example, Ferraris *et al. *[46] offered a practical, but an admittedly untested solution to the risk posed by patient co-morbidities: treat co-morbidities that raise the risk of readmission preoperatively. While intuitively a logical approach, this suggestion is more plausible under certain scenarios than others. A sufficient structure has to be in place to deliver that treatment. In case of infections, that care can exist within the hospital, but for chronic conditions, hospitals would need to possess an ambulatory care service line or have a strong connection to ambulatory care providers.

The concern over factors not modifiable by the hospital and the perceived need for continued, active post-discharge management are the types of reasons that justify integrated delivery systems and, now, the push toward accountable care organizations. Through vertical integration, integrated delivery systems are (theoretically) poised to facilitate transitions between different levels of care, and the care between inpatient, outpatient, and ambulatory care are better aligned. Accountable care organizations are to achieve the same alignment of effort toward the care of a population of patients [68]. Becoming an integrated delivery system is not exactly a fast or necessarily feasible response. Accountable care organizations function under a variety of structures, possibly tied together only through a joint financial arrangement like a bundled payment or shared information system, which is at least somewhat more feasible to develop. Alternatively, those with the extra-hospital view will undoubtedly continue to look for more effective interventions for patients they rarely see.

### Limitations

First, as is the case with all reviews, even though we searched six databases for this review it is possible we omitted some studies. One of the included databases does include grey literature, but we would assume that is the source area in which this review may be lacking. However, because we were not attempting to quantify any effect sizes, this deficit probably does not dramatically alter any of our conclusions. Second, because we are concerned with the effects of organization and environment as well as the individual- and encounter-level determinants of readmissions, we limited our investigation to US based studies. However, significant and high quality work in defining and modeling predictive readmissions has been done internationally. A cursory look at this literature concurs with our earlier assumption of consistency of patient-level and encounter characteristics internationally. For example, older age [69,70], ill health [70,71], longer length of stays [71], and prior utilization [69,71] also appear as risks in other countries. Although potentially technically challenging, cross-national comparisons may prove to be very informative.

## Summary

Despite the fact it is not always clear what is meant by a preventable (or even an early, unplanned, or avoidable) readmission, these unwanted events are not completely random occurrences. Multiple studies in various populations and with different index conditions, time frames, and reasons for readmission indicate that patients with generally worse health and greater frailty are more likely to be readmitted. However, this does not point the way to a single intervention. Furthermore, these readmissions are likely to be the target of healthcare finance reform because, regardless of definitions, methodologies exist to identify hospitals that vary according to this indicator. Unfortunately, the current identification of aberrant performance does not provide direction on how to reduce their occurrence. How hospitals will respond to these changes in reimbursement will likely be related to where they believe the problems originate within or without their own walls. Certainly, policy makers, payers, and providers (not to mention patients) have a significant stake in increased knowledge of preventable readmissions and in identification of strategies that can best reduce such occurrences. We recommend increased support for research that can address these issues. With the significantly increased focus on information technology, medical homes, and accountable care organizations, we recommend support for applied research that can provide more immediate knowledge of factors that can reduce preventable readmissions. Moreover, we call for research that both builds on understanding of the variability and gaps in current research and relies on conceptual and statistical models that begin to address the tremendous complexity of the preventable readmission.

## Competing interests

The authors declare that they have no competing interests.

## Authors' contributions

JV conceived the research question, designed the study, abstracted data, analyzed results and drafted the manuscript. LG conceived the research question, analyzed results and drafted and revised the manuscript. BO, MM, and KJ abstracted data, analyzed results and helped prepare and revise the manuscript. All authors read and approved the final manuscript.

## Authors' Information

JV was at the Texas A&M Health Science Center School of Rural Public Health during part of the preparation of this manuscript. LG is also the director of The Center for Health Organization Transformation (CHOT) is an industry-university cooperative research center (I/UCRC) funded by the National Science Foundation.

## Appendices

### Appendix 1. Example of search strategy

MEDLINE

1. All field search of hospital readmission

2. All field search of hospital readmissions

3. MESH term patient readmission

4. All field search of rehospitalization

5. #1 OR #2 OR #3 OR #4

6. All field search of risk factors

7. All field search of determinants

8. All field search of predictors

9. All field search of characteristics

10. #6 OR #7 OR #8 OR #9

11. #5 AND #10

12. All field search of unplanned

13. All field search of avoidable

14. All field search of preventable

15. All field search of early

16. #12 or #13 or #14 or #15

17. #11 AND #16

18. Limits: English. Year 2000/1/1 - 2009/12/31. Abstract

Number retrieved = 812
